# Modulation of Intestinal Phosphate Transport in Young Goats Fed a Low Phosphorus Diet

**DOI:** 10.3390/ijms22020866

**Published:** 2021-01-16

**Authors:** Joie L. Behrens, Nadine Schnepel, Kathrin Hansen, Karin Hustedt, Marion Burmester, Stefanie Klinger, Gerhard Breves, Alexandra S. Muscher-Banse

**Affiliations:** Institute for Physiology and Cell Biology, University of Veterinary Medicine Hannover, 30173 Hannover, Germany; joie.behrens@tiho-hannover.de (J.L.B.); nadine.schnepel@tiho-hannover.de (N.S.); kathrin.hansen@tiho-hannover.de (K.H.); karin.hustedt@tiho-hannover.de (K.H.); marion.burmester@tiho-hannover.de (M.B.); stefanie.klinger@gmx.net (S.K.); Gerhard.Breves.iR@tiho-hannover.de (G.B.)

**Keywords:** intestine, NaPiIIb, phosphate homeostasis, PiT1, PiT2

## Abstract

The intestinal absorption of phosphate (P_i_) takes place transcellularly through the active NaPi-cotransporters type IIb (NaPiIIb) and III (PiT1 and PiT2) and paracellularly by diffusion through tight junction (TJ) proteins. The localisation along the intestines and the regulation of P_i_ absorption differ between species and are not fully understood. It is known that 1,25-dihydroxy-vitamin D_3_ (1,25-(OH)_2_D_3_) and phosphorus (P) depletion modulate intestinal P_i_ absorption in vertebrates in different ways. In addition to the apical uptake into the enterocytes, there are uncertainties regarding the basolateral excretion of P_i_. Functional ex vivo experiments in Ussing chambers and molecular studies of small intestinal epithelia were carried out on P-deficient goats in order to elucidate the transepithelial P_i_ route in the intestine as well as the underlying mechanisms of its regulation and the proteins, which may be involved. The dietary P reduction had no effect on the duodenal and ileal P_i_ transport rate in growing goats. The ileal PiT1 and PiT2 mRNA expressions increased significantly, while the ileal PiT1 protein expression, the mid jejunal claudin-2 mRNA expression and the serum 1,25-(OH)_2_D_3_ levels were significantly reduced. These results advance the state of knowledge concerning the complex mechanisms of the P_i_ homeostasis in vertebrates.

## 1. Introduction

The regulation of the mineral homeostasis in vertebrates has been the subject of research for decades. While knowledge about the balance of calcium (Ca) is well advanced, there are still great uncertainties concerning phosphate (P_i_). P_i_ is an essential component of organisms and is involved in many life processes, including energy metabolism, pH buffering and signal transduction. As disturbances in P_i_ homeostasis cause serious pathologies, the study of P_i_ balance and its underlying mechanisms including the intestinal absorption are of particular interest. Intestinal P_i_ absorption from the diet occurs either transcellularly through active transport processes or through paracellular pathways by diffusion. Research on different species has provided inconsistent results regarding the intestinal distribution and regulation of P_i_ transporters. In mice, it was found that active P_i_ transport occurs predominantly in the ileum through an Na^+^-dependent process [1]. Experiments with rats showed that the active P_i_ transport is most abundant in the duodenum and decreases continuously along the intestinal axis towards the ileum [2]. In adult sheep and growing goats, the ileum has been identified as the main site of active P_i_ transport [3,4]. The transcellular P_i_ absorption in the intestine is mediated by secondary active Na^+^-coupled P_i_ transporters, which are driven by an Na^+^-gradient that is generated by the basolateral localised enzyme Na^+^/K^+^-adenosine triphosphatase (Na^+^/K^+^-ATPase) [5].

The NaPi cotransporter type IIb (NaPiIIb, SLC34A2) was found to be expressed in the intestine and in the lung [6], whereas the NaPi cotransporters type III (PiT1, SLC20A1 and PiT2, SLC20A2) are expressed in various tissues including the intestines, lungs, liver and bones [7]. The expression of both transporter types, NaPiIIb and PiT1/2, is regulated by the supply of phosphorus (P) in different small intestinal segments across the species. Upregulation of Na^+^-dependent P_i_ transport by dietary P reduction has been described for the duodenal segment of sheep [4,8]. In this intestinal segment, however, no NaPiIIb could be detected [9], so that the underlying molecular mechanism remained unclear. As in monogastric species, NaPiIIb-mediated P_i_ transport in the jejunum has been shown in goats [4,10,11], which was stimulated by P depletion [9]. In rats with a P deficiency, the expression of PiT1/2 increased in the duodenum and jejunum, while the expression of PiT2 also increased in the ileum [12]. In addition, in has been shown in monogastric species that 1,25-dihydroxy-vitamin D_3_ (1,25-(OH)_2_D_3_) modulates *NaPiIIb* as well as *PiT1* and *PiT2* expression after binding to the vitamin D receptor (VDR) [13,14]. Furthermore, 1,25-(OH)_2_D_3_ is the biologically active form of the vitamin D that is synthesised by the conversion of 25-hydroxy-vitamin D_3_ (25-(OH)D_3_), mediated by a renal enzyme. The underlying mechanism of the modulation of the Na^+^-dependent P_i_ absorption in the intestines differs depending on the species, since NaPiIIb in mice with P deficiency was regulated without involvement of the vitamin D axis [15], and NaPiIIb in goats, despite reduced 1,25-(OH)_2_D_3_ levels, remained unchanged [3].

The mechanism of intracellular P_i_ transport and the basolateral export of P_i_ is still largely unknown. It is assumed that the xenotropic and polytropic retrovirus receptor 1 (XPR1) is involved in the basolateral export of P_i_ and in P_i_ sensing in epithelial cells [16,17]. The passive intestinal P_i_ absorption takes place according to the electrochemical gradient via tight junction (TJ) proteins, which form selective barriers in the paracellular space between epithelial cells, limiting transepithelial movement of ions, solutes and water [18]. An important group of TJ is represented by the claudins, which can be divided into sealing and permeability-mediating members [19]. It has been found that claudin-2, claudin-12 and claudin-15, which belong to the latter group, form cation-selective, water-permeable pores [20,21]. Claudin-1 is believed to have sealing properties that contribute to the maintenance of the epithelial barrier and epithelial homeostasis [22,23]. Another protein associated with the TJ is the ZO-1, a scaffold protein located at the cytosolic side of the cell membrane, providing structural stability by cross-linking between TJ proteins and the cytoskeleton [24,25]. Occludin is another TJ protein known to mediate the paracellular permeability of macromolecules [26]. Besides the TJ, the adherens-junctions (AJ) are also part of the junctional complex, including cadherin-17, which provides structural stability and integrity of the epithelia [27]. TJ and AJ proteins are modulated by various mediators, such as growth hormones, cytokines and bacterial toxins. A regulation of paracellular mineral transport by 1,25-(OH)_2_D_3_ has been demonstrated for claudin-2, claudin-12 and cadherin-17 [21,28]. Little is known about the paracellular P_i_ transport route and the TJ proteins involved in its mediation.

In order to gain insight into the mechanisms of active and passive P_i_ transport in the small intestines of growing goats and its regulation by dietary P reduction, functional ex vivo investigations of the duodenal and ileal epithelia in Ussing chamber experiments and molecular determinations of the small intestinal epithelia were carried out. In this study, low P feeding had no effect on the P_i_ transport rate in the duodenum and ileum. The presence of PiT1 and PiT2 throughout the small intestine was shown, while NaPiIIb expression was restricted to the jejunum and ileum. Both ileal PiT1 and PiT2 mRNA expression increased, whereas protein expression of PiT1 decreased significantly and that of NaPiIIb showed a downward trend in the ileum. Claudin-2 mRNA decreased in the mid jejunum by the dietary P reduction. The regulation of P_i_ homeostasis in vertebrates is of great complexity and the underlying mechanisms vary widely between species. Therefore, this requires further research for a better understanding.

## 2. Results

### 2.1. Intake, Body Weight and Daily Body Weight Gain of Young Goats Fed a Low P Diet

The animals were clinically healthy during the rearing period and the experimental period. The daily energy and Ca supply of both feeding groups and the P supply of the control group were in accordance with the recommendations of the Society of Nutrition Physiology (GfE) for young ruminating goat kids [29]. The daily intake of dry matter (DM), P and Ca from the concentrate was monitored individually for each animal and from the wheat straw estimated by group averages for each animal. The results are summarised in Table 1. The daily body weight gain and the final body weight of goats fed a reduced P diet were significantly reduced compared to goats in the control group (Table 2).

### 2.2. Concentrations of P_i_ and Ca in Body Fluids of Young Goats Fed a Low P Diet

In goats fed a reduced P diet, P_i_ concentrations decreased significantly in all body fluids examined, while Ca concentrations increased significantly in all body fluids with the exception of abomasal fluid, where the increase was not significant (Table 3). The 25-(OH)D_3_ concentrations increased significantly by 18.7% (*p* = 0.031), while the 1,25-(OH)_2_D_3_ concentrations in the serum of the reduced P feeding group decreased significantly by 27.5% (*p* = 0.035).

### 2.3. Flux Rates of P_i_ Across the Duodenal and Ileal Epithelia of Young Goats Fed a Low P Diet

**Figure 1:**
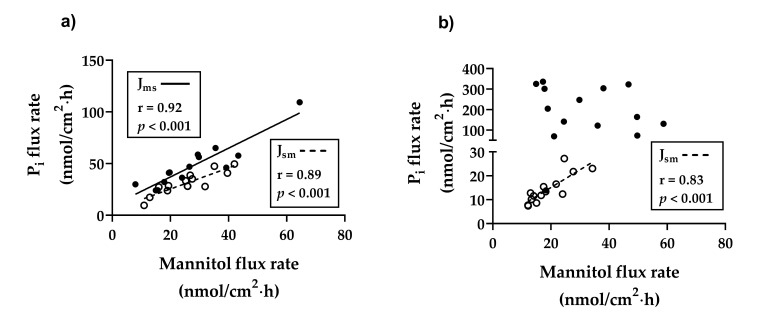
Linear regression of the unidirectional mucosal to serosal (J_ms_; ⚫, **───**) or serosal to mucosal (J_sm_; ⚪, **- - - -**) flux rates of inorganic phosphate (P_i_) with the corresponding mannitol flux rates in duodenum (**a**) (J_ms_ P_i_ = (1.39 ± 0.17), J_ms_ mannitol = (9.19 ± 5.33); J_sm_ P_i_ = (1.02 ± 0.15), J_sm_ mannitol = (4.88 ± 4.21)) and ileum (**b**) (J_ms_ P_i_ = −(1.40 ± 2.10), J_ms_ mannitol = (240.9 ± 72.56), *r* = 0.189, *p* = 0.5163; J_sm_ P_i_ = (0.74 ± 0.14), J_sm_ mannitol = (0.28 ± 2.87)) from goats fed different phosphorus supply.

The P_i_ net flux rates (J_net_) were significantly higher in the ileum (average of 182.42 nmol/cm^2^∙h) than in the duodenum (16.67 nmol/cm^2^∙h) (*p* < 0.001). The mannitol J_net_ flux rates were also significantly higher in the ileum (average of 12.65 nmol/cm^2^∙h) than in the duodenum (2.12 nmol/cm^2^∙h) (*p* < 0.05). In both the duodenal and the ileal segment, the P_i_ flux rates remained unaffected by dietary P reduction (Table 4). The addition of Na^+^-arsenate led to a decrease in P_i_ J_net_ by 189% in the duodenum and of 79% in the ileum with no significant differences between both groups. The addition of TAP resulted in a decrease in P_i_ J_net_ of 148% in the duodenum and of 2.54% in the ileum with no significant differences between the feeding groups (data not shown). For both intestinal segments, significant correlations between P_i_ J_sm_ and mannitol J_sm_ were shown by linear regression (Figure 1a,b). Another positive correlation could be shown between P_i_ J_ms_ and mannitol J_ms_ in the duodenum (Figure 1a), while no correlation was found between P_i_ J_ms_ and mannitol J_ms_ in the ileum (Figure 1b).

### 2.4. Electrophysiological Parameters of Duodenal and Ileal Epithelia of Young Goats Fed a Low P Diet

**Figure 2:**
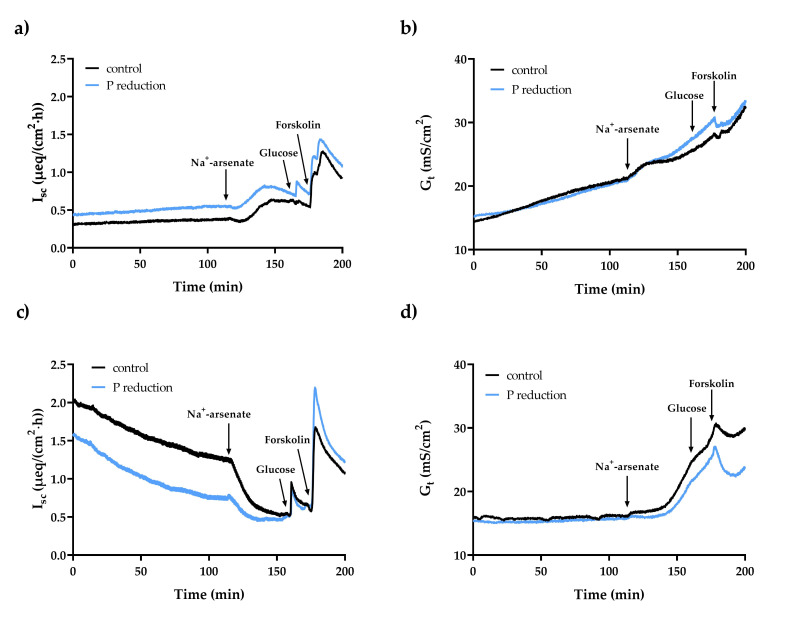
Effect of adding 5 mmol/L Na^+^-arsenate to the mucosal side of duodenal epithelia on the short-circuit current (I_sc_) (**a**) and the tissue conductance (G_t_) (**b**). Effect of adding 5 mmol/L Na^+^-arsenate to the mucosal side of ileal epithelia on the short-circuit current (I_sc_) (**c**) and the tissue conductance (G_t_) (**d**).

**Figure 3:**
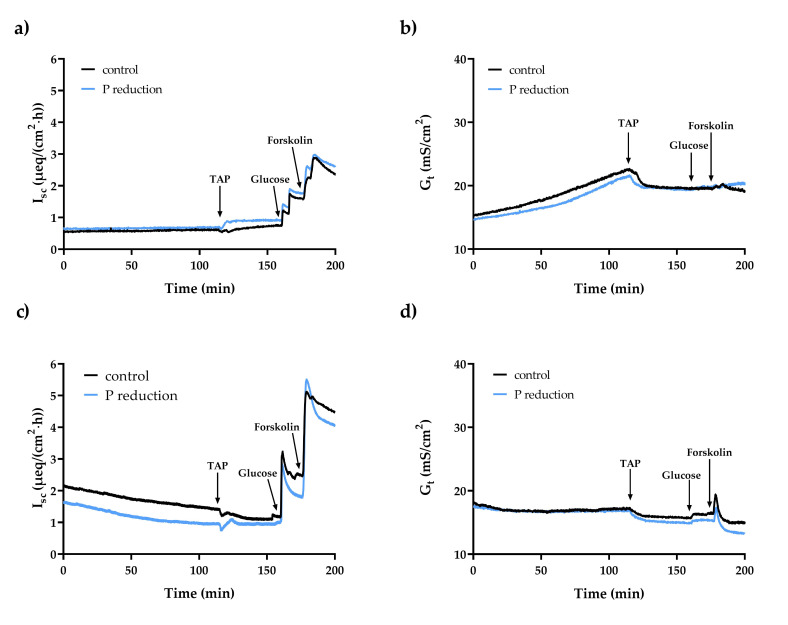
Effect of adding 20 mmol/L TAP to the mucosal side of duodenal epithelia on the short-circuit current (I_sc_) (**a**) and the tissue conductance (G_t_) (**b**). Effect of adding 20 mmol/L TAP to the mucosal side of ileal epithelia on the short-circuit current (I_sc_) (**c**) and the tissue conductance (G_t_) (**d**).

The basal I_sc_ and G_t_ were neither in the duodenum nor in the ileum in young goats affected by the reduced P feeding regime (Table 5). In duodenal epithelia, the addition of Na^+^-arsenate led to an increase in I_sc_ (control: *p* < 0.01; P reduction: *p* < 0.001) and G_t_ (control and P reduction: *p* < 0.01) (Figure 2a,b). In ileal epithelia, the I_sc_ decreased after Na^+^-arsenate was added (control: *p* < 0.01; P reduction: *p* = 0.055), while the G_t_ reacted with a delayed increase (control: *p* < 0.01; P reduction: *p* < 0.05) (Figure 2c,d). The addition of TAP resulted in a transient decrease in I_sc_ in both intestinal segments (duodenum: control and P reduction: *p* < 0.01; ileum: control and P reduction: *p* < 0.05), followed by a slight increase in the duodenum of the reduced P feeding group (Figure 3a,c). The G_t_ decreased in both intestinal segments after the addition of TAP (duodenum: control: *p* < 0.01, P reduction: *p* < 0.05; ileum: control and P reduction *p* < 0.01) (Figure 3b,d). The changes in I_sc_ (∆I_sc_) and G_t_ (∆G_t_) after adding the inhibitors showed no significant group differences. All data are summarised in Table 6.

### 2.5. Intestinal mRNA Expression of Na^+^/K^+^-ATPase, NaPiIIb, PiT1, PiT2, VDR, XPR1, Cadherin-17, Claudin-1, Claudin-2, Claudin-12, Claudin-15, Occludin and ZO-1 in Young Goats Fed a Low P Diet

**Figure 4:**
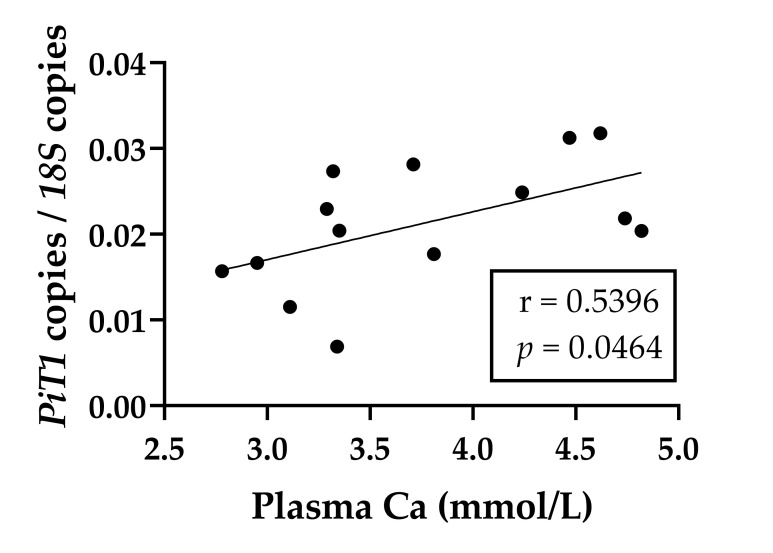
Linear regression of ileal *PiT1* mRNA expression normalised to *18S* with plasma Ca (*PiT1* mRNA = (0.0056 ± 0.0025), plasma Ca = (0.0003 ± 0.0096)) of goats fed different phosphorus supply.

In this section, the results are only described in detail if there was at least a tendency for group differences (*p* < 0.1). For the isolated RNA from intestinal epithelia used for quantitative PCR, the mean RNA integrity numbers with their standard errors were determined in the duodenum (8.11 ± 0.29), mid jejunum (8.28 ± 0.28) and ileum (8.91 ± 0.26). The claudin-2 mRNA expression was significantly decreased in the mid jejunal epithelia of the reduced P feeding group. The *PiT1* and *PiT2* mRNA expression was significantly increased in the ileal epithelia of the reduced P feeding group. *NaPiIIb* in the duodenal epithelia and both cadherin-17 and *ZO-1* in the ileal epithelia tended to be higher in the reduced P feeding group. All data are summarised in Table 7. The mRNA expression of *PiT1* correlated positively with plasma Ca concentrations (Figure 4).

### 2.6. Intestinal Protein Expressions of Na^+^/K^+^-ATPase, NaPiIIb and PiT1 in Ileal Epithelia of Young Goats Fed a Low P Diet

**Figure 5:**
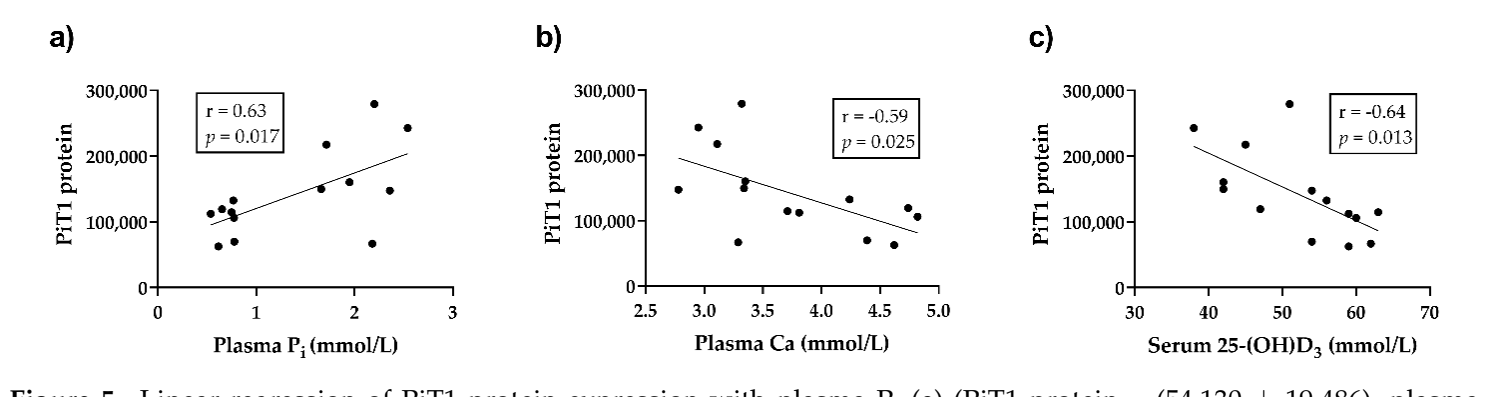
Linear regression of PiT1 protein expression with plasma P_i_ (**a**) (PiT1 protein = (54,130 ± 19,486), plasma P_i_ = (66,268 ± 30,620)), plasma Ca (**b**) (PiT1 protein = (−56,174 ± 21,950), plasma Ca = (352,119 ± 83,565)) and serum 25-(OH)D_3_ (**c**) (PiT1 protein = (−5133 ± 1765), serum 25-(OH)D_3_ = (409,943 ± 93,332)).

The protein expression of PiT1 decreased significantly and that of NaPiIIb showed a downward trend in the ileal epithelia of the reduced P feeding group, while no changes were found in the protein expression of Na^+^/K^+^-ATPase (Table 8). The PiT1 protein expression (Figure 5a), the Na^+^/K^+^ATPase protein expression (Na^+^/K^+^ATPase protein = (112,467 ± 44,092), plasma P_i_ = (1,820,969 ± 69,285), *r* = 0.59, *p* = 0.025) and the NaPiIIb protein expression (NaPiIIb protein = (183,940 ± 92,068), plasma P_i_ = (298,931 ± 144,673), *r* = 0.50, *p* = 0.07) showed a trend regarding positive correlation with the plasma P_i_ concentrations, while a negative relationship was detected between the PiT1 protein expression and both the plasma Ca concentrations (Figure 5b) and the serum 25-(OH)D_3_ levels (Figure 5c).

## 3. Discussion

The aim of this study was to investigate the influence of dietary P reduction on transepithelial P_i_ transport at a functional and molecular level along the small intestinal axis of young goats. Changes in mineral homeostasis, such as hypophosphataemia and hypercalcemia are well known in P-deficient animals [30,31,32,33] and are also reflected in other examined body fluids. Hypercalcemia could result from increased resorption of Ca-P_i_ compounds from the skeleton, in order to compensate for the inadequate supply of P [34].

In addition to hypophosphataemia, which is usually associated with reduced feed consumption [35,36,37,38], the described hypercalcaemia could lead to a depression in feed intake [39,40,41] and thus may have caused the reduction in feed intake and final body weight. Although the underlying control mechanism of decreased feed intake due to low P diets is still unclear, it is believed that hypophosphataemia leads to a central nervous system disorder, as human patients with severe hypophosphataemia suffered from confusion and loss of appetite, which could be resolved by administration of P_i_ [42]. It is assumed that hypercalcaemia impairs the contractility of the smooth muscles of the gastrointestinal tract and thus causes digestive disorders along with reduced appetite [43,44,45]. In addition, a P_i_ deficiency in the forestomach of ruminants could lead to reduced microbial fermentation and a lower digestibility of organic matter. However, the P_i_ concentration measured in the ruminal fluid was well above the minimal requirement of 0.7–2.6 mmol/L and can therefore be excluded as a cause of the reduced body weight gain [46,47,48].

In monogastric species, e.g., pigs, rats and chickens, it was demonstrated that 1,25-(OH)_2_D_3_ levels increased due to low P (and/or low Ca) feeding [49,50,51], while in small ruminants this was only the case in response to a depletion of Ca [3], but not of P [52,53]. One reason for the decreased 1,25-(OH)_2_D_3_ levels in the present study could be the particularly pronounced hypercalcaemia, since experiments with rats have shown that elevated blood Ca concentrations down-regulate the 1,25-(OH)_2_D_3_ levels [54,55].

Both P depletion [4,9,45] and 1,25-(OH)_2_D_3_ [10,56,57,58] stimulate P_i_ absorption in the small intestines of various species, including ruminants. The results of several studies with rodents have indicated that increased intestinal P_i_ absorption is due to stimulated intestinal *NaPiIIb* expression, which is mediated by increased 1,25-(OH)_2_D_3_ [14,59]. However, in VDR- or 1α hydroxylase-knockout (KO) mice fed a reduced P diet, the expression of *NaPiIIb* increased as in wild type animals, thus without involvement of the vitamin D axis, demonstrating the existence of a vitamin D-independent mechanism for upregulation of intestinal P_i_ transport [15]. Earlier experiments with P-depleted mice showed that the upregulation of renal *NaPiIIa* was mediated by increased expression of the transcription factor µE3 (TFE3), which promotes the expression of the transporter gene via P_i_ response elements in its promoter sequence [60]. Similar mechanisms are considered to be involved in mediating a diet-induced upregulation of *NaPiIIb* in the intestine of mice [61] and could, therefore, possibly be applied to ruminants.

In the present study, the duodenal P_i_ flux rates were relatively low compared to the jejunum [62] and ileum and were in a similar range to duodenal P_i_ transport from previous experiments with goats [3,4], indicating that the duodenum is of minor importance for intestinal P_i_ absorption. The applied feeding regime had no effect on P_i_ transport in the duodenum. This is in line with previous studies in goats that demonstrated increased P_i_ transport due to low P feeding in the jejunum, while duodenal P_i_ transport remained unchanged. In addition, it was shown that the upregulation of active jejunal P_i_ transport is mediated by an increase in NaPiIIb protein expression [9]. Since NaPiIIb is practically non-existent in the duodenum of ruminants and is mainly expressed in the jejunum and ileum [3,63], an upregulation of the active P_i_ transport induced by low P feeding and mediated by an increase in NaPiIIb, as mentioned above for the jejunum [9], can be excluded for the duodenum of growing goats. In fact, no upregulation of *NaPiIIb* due to reduced P feeding was demonstrated at the mRNA level in the duodenum and jejunum and neither at the mRNA nor at the protein level in the ileum, suggesting that neither the ileum nor the duodenum is a target of P deficiency induced upregulation of *NaPiIIb*. An upregulation of jejunal NaPiIIb at the protein level without impairing the transcription rate, as demonstrated in previous studies [9], cannot be ruled out and is very likely. In addition, an extensive part of the duodenal P_i_ transport in the Ussing chamber experiments occurred via the paracellular pathway, as indicated by the positive correlation between J_ms_ flux rates of P_i_ and the corresponding mannitol flux rates. Under in vivo conditions, there is an electrical gradient across the intestinal epithelium (negative mucous membrane) [64], which could potentially drive the passive P_i_ transport even if the plasma P_i_ exceeds the lumen P_i_ [65]. Since the electrical gradient is eliminated in Ussing chamber experiments, the actual extent of passive P_i_ transport under physiological conditions remains unclear.

Relatively high P_i_ flux rates in the ileum are in agreement with the results of previous experiments in which the ileum was identified as the intestinal segment with the highest P_i_ transport rate in ruminants [3]. Due to the short length and the localisation of the ileum at the end of the small intestine, as well as the physiological properties of the chymus, which contains lower concentrations of P_i_ [66], P_i_ absorption can only be mediated by active transport. The missing correlation between J_ms_ flux rates of P_i_ and the corresponding mannitol flux rates confirmed that the P_i_ absorption in the ileum was mainly via the transcellular pathway. Another group of P_i_ transporters (SLC20) that contribute to active P_i_ transport includes the NaPi-cotransporters PiT1 and PiT2. The mRNA expression of *PiT1* and, for the first time in goats, *PiT2* was found in all examined intestinal segments. While *PiT1* and *PiT2* mRNA remained unchanged in the duodenum, both increased in the ileum due to dietary P reduction. Paradoxically, it was found that ileal PiT1 protein expression was downregulated, which is reflected in unchanged ileal P_i_ transport. In rats, administering 1,25-(OH)_2_D_3_ apparently upregulates intestinal *PiT2* [13], while P depletion upregulates both PiT1 and PiT2 [12]. The underlying mechanism of the regulation of PiT1 and PiT2 has not yet been conclusively clarified and is the subject of current research, considering P_i_-sensing and the post-translational modulation of their activity by extracellular P_i_ [67,68]. The fact that *PiT1* mRNA expression correlates positively, while its protein expression correlates negatively with plasma Ca, indicates complex regulative mechanisms by the mineral homeostasis. Moreover, a direct effect of plasma P_i_ on the protein expression of PiT1 cannot be ruled out since a positive correlation was found. However, it is also possible that 25-(OH)D_3_ is involved in the modulation of PiT1, as it negatively correlates with PiT1 protein expression.

Adding Na^+^-arsenate led to a significant decrease in duodenal and ileal P_i_ transport rates, which can be explained by competitive inhibition of the active P_i_ transporters PiT1 and PiT2 in both segments and also NaPiIIb in the ileum [5]. TAP is known as a blocker of TJ proteins [69] and inhibits the paracellular transport of P_i_ in a yet unknown way. Adding TAP led to an enormous reduction in P_i_ transport in the duodenum, since paracellular transport is particularly extensive here, while the reduction in the ileum was negligible, due to the minor role played by the paracellular pathway of P_i_ in the ileum.

By passing an external current through the epithelium, the potential was clamped to zero in order to eliminate the passive transepithelial driving force, generated by the spontaneous electrical potential across the epithelium. This current is equivalent to the algebraic sum of electrogenic ion movement by active transport and is referred to the short-circuit current (*I*_sc_) [70]. A positive value indicates the transport of mainly ions with a positive charge in a serosal direction or negatively charged ions in the opposite direction. The electrophysiological parameters remained unchanged in both the duodenum and the ileum by the dietary P reduction, which in the case of I_sc_ could be due to the unchanged P_i_ transport rates in the intestinal segments due to the applied feeding regime. For the G_t_, it can be concluded that the reduced P feeding had no influence on the integrity and therefore on the permeability of the tissues.

The I_sc_ value measured for the duodenum is similar to those measured in previous studies with sheep and goats [4,62]. It is known that PiT1 and PiT2 preferentially transport monovalent P_i_ and that according to the Na^+^:P_i_ stoichiometry of 2:1, with each P_i_-anion, a positive charge is transported in the serosal direction [5,12,71], which results in a positive I_sc_ value. That I_sc_ is highest in the ileum has already been shown in sheep and goats [4,72] and indicates either higher net absorption of cations or higher net secretion of anions than in upper intestinal segments. This can most likely be explained by the greater abundance of PiT1 and PiT2 in the ileum, which shifts a positive charge to the serosal side of the epithelium with each P_i_-anion. In addition, NaPiIIb is expressed in the ileum, which participates in active P_i_ transport and preferentially transports divalent P_i_, whereby again, one positive charge is moved to the serosal side of the epithelium [71]. Therefore, the sum of the charge shifts can result in a higher I_sc_ in the ileum compared to the duodenum. The G_t_ values measured in the duodenum and ileum in the present study are similar to those in previous studies with sheep and goat intestine and confirm the integrity of the epithelium over the duration of the experiment [4,62,72].

The increased I_sc_ in the duodenum after the adding Na^+^-arsenate indicates an ion current through the epithelium, which could be caused by PiT1 activity [73] or by passive diffusion of cations to the serosal side following the chemical gradient. In the ileum, Na^+^-arsenate causes a significant reduction in I_sc_, which can be explained by the competitive inhibition of NaPiIIb, PiT1 and PiT2 [5], which reduces the movement of positive charge to the serosal side. As a TJ blocker, TAP slightly lowered I_sc_ in the duodenum and more clearly in the ileum for a short time, followed by an increase in the duodenum of the P-reduced feeding group, which indicates an induced charge shift across the epithelia after inhibition. Moreover, the blocking of TJ proteins by TAP resulted in significantly reduced G_t_ values, which indicates a reduced paracellular permeability in the duodenum and ileum.

In order to investigate a potential involvement of the vitamin D axis in the regulation of P_i_ transport in growing goats it was of interest to investigate whether the VDR, a ligand-dependent transcription factor [74] is modulated by the applied feeding regime. In VDR KO mice, hypocalcaemia and hypophosphataemia occurred, accompanied by skeletal changes as rickets and osteomalacia. The elimination of the vitamin D axis at the receptor level resulted in decreased intestinal Ca absorption, which led to secondary hyperparathyroidism. The rise in the parathyroid hormone, in turn, caused hypophosphataemia due to increased renal P_i_ secretion [75]. In P-depleted rats, VDR was upregulated in the intestine [76]. Previous studies in P-depleted goats addressed the receptor-binding affinity and found it to be increased in lactating [77], but not in growing goats [32]. In the present study, the *VDR* mRNA expression in the duodenum, jejunum and ileum did not change due to the applied feeding regime, which supports the assumption that the VDR regulation is not relevant for the upregulation of intestinal P_i_ absorption in P-depleted growing goats.

For a better understanding of the transcellular P_i_ pathway, it is of interest to explain not only the apical P_i_ uptake into the enterocytes, but also the basolateral P_i_ export. Experiments in cell cultures showed that the depletion of XPR1 induced a decrease in P_i_ export and that reintroduction of various XPR1 proteins resolved this defect. From these results, it was concluded, that XPR1 is a P_i_ exporter in vertebrates [17]. In the present study, *XPR1* mRNA expression could be found in all examined intestinal segments and the mRNA levels remained unchanged, which indicates that XPR1 mediates the basolateral P_i_ export in growing goats and is not influenced by P reduced feeding.

In standard or high P diets, passive transepithelial P_i_ transport, driven by electrochemical gradients, has been shown to be the predominant P_i_ pathway in rodents, as the luminal P_i_ concentration exceeded that of the plasma severalfold, providing a chemical gradient for passive transport [65]. In order to investigate a potential modulation of paracellular P_i_ absorption by reduced dietary P, the present study focused not only on proteins involved in active P_i_ absorption, but also on TJ proteins, which are potentially involved in the mediation of the paracellular P_i_ pathway. TJ discriminate ions by charge and size [78] and appear to preferentially transport the monovalent P_i_ species, which tends to be more abundant at a rather acidic pH [65]. This supports the assumption that in growing goats the paracellular P_i_ uptake is particularly extensive in the duodenum, where the pH of the chymus is lower than in distally located intestinal segments, as has been shown in sheep [79].

Little is known about the involvement of the TJ proteins mentioned above in the mediation of paracellular P_i_ transport. Previous research in goats and VDR-KO mice revealed a 1,25-(OH)_2_D_3_-dependent regulation of members of the claudin family including claudin-2, which plays an important role in paracellular Ca transport [21,72]. Thus, down-regulation of jejunal claudin-2 expression in the present study is assumed to be a consequence of diminished 1,25-(OH)_2_D_3_ levels in P-depleted goats, although no correlation could be found between jejunal claudin-2 mRNA expression and serum 1,25-(OH)_2_D_3_.

Both transcellular and paracellular P_i_ transport in the vertebrate intestine are not yet fully understood. The regulatory mechanisms differ between species and are the subject of ongoing research. The results of this study contribute to a better understanding of the transepithelial P_i_ signalling pathway and the regulation of P_i_ transport by dietary P depletion.

## 4. Materials and Methods

### 4.1. Animals and Feeding Regimes

The experimental conditions of animal treatment and feeding were approved by the Animal Welfare Officer of the University of Veterinary Medicine Hannover, Hannover, Germany and were in accordance with the German Animal Welfare Law (permit number: 33.19-42502-04-19/3076; Lower Saxonian State Office for Consumer Protection and Food Safety (LAVES); 15 March 2019).

Fourteen male Coloured German goats of about one week of age were randomly assigned by weight-matched pairs into two groups of seven animals each and kept in stable compartments of approximately eight square metres bedded with sawdust. During the rearing, the animals were fed with a commercially available milk replacer and wheat straw ad libitum. From the 6th week of life and over a period of four weeks, the goats were gradually weaned and adapted to the pelleted control diet. Subsequently, the control diet in one group was gradually replaced by a P-reduced experimental diet within five days. The pelleted concentrate was offered to the goats individually twice daily (55 g/kg^0.75^/d), while straw was offered in group feeding (25% of the concentrate weight). Water was available ad libitum. The feeds offered and refused were measured daily to determine the mean intake per animal. The amount of feed was adjusted weekly to the weight of the animals.

### 4.2. Diets

The DM, crude ash, CP, crude fat and crude fibre content of the feed was determined by Weende analysis this being the standard method of the Association of German Agricultural Investigation and Research Centre (VDLUFA) [80]. The content of acid-detergent fibre and neutral-detergent fibre was determined using the Van Soest method and corrected to organic matter (ADFom; aNDFom) [81]. The control diet contained 0.42% P, while the reduced P diet contained 0.10% P. The diets were isoenergetic (approximately 12.9 MJ ME/kg DM) and contained comparable amounts of Ca (approximately 12.5 g/kg DM). The components and composition of the diets are summarised in Table 9.

### 4.3. Intestinal Tissue and Body Fluid Sampling

After an experimental period of five to seven weeks, one animal per day was slaughtered by exsanguination after captive bolt stunning in a group-alternating manner. Blood and saliva samples were taken shortly before the slaughter. Blood samples of 9 mL each were obtained by puncturing the external jugular vein with lithium heparin- and EDTA-coated syringes and serum syringes (Sarstedt AG & Co. KG, Nümbrecht, Germany). The plasma separation was carried out by centrifugation at 2000× *g* for 15 min at room temperature (RT). Saliva samples were collected using the Salivette^®^ (Sarstedt). For this purpose, a cotton swab was placed in the cheek pouch for 2 min using an artery clamp, before it was centrifuged in the test tube at 1000× *g* for 2 min at RT. Blood and saliva samples were stored at −20 °C. Intestinal segments (duodenum, mid jejunum and ileum) were exenterated within 5 min after death. For the Ussing chamber experiments, duodenal and ileal segments were rinsed with ice-cold saline (0.9%, *w*/*v*), opened along the mesenteric line and kept in a modified glucose-containing Krebs–Henseleit buffer solution with carbogen aeration (95% O_2_, 5% CO_2_) for further preparation. For RNA isolation and crude membrane preparation, the tunica mucosa of all intestinal segments was stripped, frozen in liquid nitrogen and stored at −80 °C.

### 4.4. Ussing Chamber Experiments

Ussing chamber experiments provide insight into transepithelial transport processes with regard to flux rates and electrophysiological parameters. The experiments were conducted with duodenal and ileal tissue samples using twelve chambers for each segment. After stripping off the tunica serosa and tunica muscularis, the remaining tunica mucosa was mounted between the two halves of the incubation chambers with an exposed serosal area of 1.0 cm^2^, dividing each chamber into a mucosal and serosal compartment. Both sides of the tissue were incubated with 10 mL of 38 °C warm buffer solution, which was continuously aerated with carbogen and maintained at pH 7.4. Both the serosal and the mucosal buffer solutions contained (mmol/L) 113.6 NaCl, 5.4 KCl, 0.2 HCl (1n), 1.2 MgCl_2_·6H_2_O, 21.0 NaHCO_3_, 1.2 CaCl_2_·2H_2_O, 1.2 Na_2_HPO_4_·2H_2_O, 1.2 mannitol and 0.01 indomethacin. The serosal buffer additionally contained (mmol/L) 10.0 glucose, 6.0 Na^+^-gluconate and 7.0 HEPES, while 6.0 NaOH (1n) and 20.0 HEPES were added to the mucosal buffer. Towards the end of the experiment, Na^+^-arsenate (5 mmol/L) or 2,4,6-triaminopyrimidine (TAP) (20 mmol/L) were added to the mucosal side of four chambers each. As a competitive inhibitor of Na^+^-dependent P_i_ transport, Na^+^-arsenate was used to investigate the transcellular P_i_ pathway [82]. TAP was used to investigate the paracellular P_i_ pathway as it blocks TJ proteins [83] and is supposed to inhibit paracellular P_i_ transport. The remaining four chambers were time controls to which no inhibitors were added.

#### 4.4.1. Flux Rate Studies

The investigation of the transepithelial flux rates of P_i_ in the duodenum and ileum was based on the radioisotope tracer technique and was conducted in accordance with established protocols using ^32^P und ^3^H(-mannitol) as radio isotopic tracers [4]. ^32^P was used to trace the total transepithelial P_i_ transports, while (^3^H-) mannitol marked the paracellular P_i_ transport [84]. After an equilibration time of 5–10 min, 148 kBq of the respective radioisotope was added to each chamber either to the serosal or mucosal side. For a period of 60 min, samples were taken from the unlabelled side at 15 min intervals and immediately replaced by equal volumes of the respective buffer solution. Additionally, samples were taken from the radioactively labelled side at the beginning and at the end of the experiment. The sampling before and after adding of inhibitors was performed to obtain information about the respective proportion of the transcellular and paracellular P_i_ transport on total P_i_ transport. A liquid scintillation counter (Packard BioScience Company, Meriden, CT, USA) was used to determine the radioactivity of the samples. Without electrochemical gradients, unidirectional flux rates of P_i_ and mannitol from mucosal to serosal (J_ms_) and in the opposite direction from serosal to mucosal (J_sm_) were calculated from the appearance rate of the respective tracer on the unlabelled side using standard equations [85]. The net flux rates (J_net_) were determined by subtracting J_sm_ from J_ms_ of paired tissues. 

#### 4.4.2. Electrophysiological Studies

Using a computer-controlled voltage clamp device (Mussler Scientific Instruments, Aachen, Germany), the potential differences, tissue conductance (G_t_) and short circuit currents (I_sc_) were continuously monitored. The experiments were carried out under short-circuited conditions. The basal values for G_t_ and I_sc_ were determined as mean values over the time before adding the inhibitor. In order to examine the effects of Na^+^-arsenate or TAP on the transepithelial transport processes in the duodenum and ileum, changes in G_t_ and I_sc_ were observed after the addition. Tissue viability was monitored by continuously recording G_t_ and I_sc_ and by adding glucose (10 mmol/L) to the mucosal side and forskolin (0.01 mmol/L) to the serosal of all chambers at the end of the experiments. The change in I_sc_ in response to the addition of glucose and forskolin indicated epithelial viability and integrity.

### 4.5. Biochemical Determinations

Inorganic P_i_ and Ca concentrations were determined colorimetrically in plasma, saliva, ruminal and abomasal fluids by standard spectrometric methods [86,87] (inter-assay CV 4.9% (P_i_) and 5.7% (Ca); intra-assay CV 1.4% (P_i_) and 3.8% (Ca)), respectively. Serum concentrations of 25-(OH)D_3_ and 1,25-(OH)_2_D_3_ were measured using HPLC or a competitive ELISA (Immundiagnostik AG, Bensheim, Germany).

### 4.6. RNA Isolation and Reverse Transcription

Using the RNeasy^®^ Plus Mini-Kit (Qiagen GmbH, Hilden, Germany), total RNA was isolated in accordance with the manufacturer’s protocol. The concentration and integrity of the isolated RNA were measured spectrophotometrically using the NanoDrop One (Thermo Fisher Scientific Inc., Waltham, MA, USA) and an RNA 6000 nanoassay for an Agilent 2100 Bioanalyzer (Agilent Technologies, Böblingen, Germany). In accordance with the manufacturer’s protocol, 200 ng of the isolated RNA was reverse-transcripted, using TaqMan™ Reverse Transcription Reagents including random hexamer and oligo dT primers (Applied Biosystems Inc., Thermo Fisher Scientific, Waltham, MA, USA).

### 4.7. Intestinal mRNA Expression of Na^+^/K^+^-ATPase, NaPiIIb, PiT1, PiT2, VDR, XPR1, Cadherin-17, Claudin-1, Claudin-2, Claudin-12, Claudin-15, Occludin and ZO-1

In order to quantify the expression abundances of *18S*, claudin-2 and claudin-12 in duodenal, mid jejunal and ileal epithelia, caprine-specific TaqMan™ primers and probes were purchased from TIB MOLBIOL (Berlin, Germany) (Table 10). Reaction mixtures of 20 µL contained TaqMan™ Universal Master Mix (Applied Biosystems Inc.), 16 ng reverse-transcripted complementary DNA (cDNA), 300 nmol/L specific primers and 100 nmol/L specific probe. PCR product amplification (2 min at 50 °C; 10 min at 95 °C; 40 cycles of 15 s at 95 °C and 1 min at 60 °C) and analysis were conducted using a real-time PCR cycler (CFX96TM; Bio-Rad Laboratories GmbH, Munich, Germany). The expression of *NaPiIIb*, *PiT1*, *PiT2*, *Na^+^/K^+^-ATPase*, *XPR1*, claudin-1, claudin-15, cadherin-17, occludin, *ZO-1* and *VDR* in the duodenal, mid jejunal and ileal epithelia were determined using SYBR Green^®^ PCR assays. Specific primers for expression abundance determinations were in the case of *NaPiIIb*, *PiT1*, cadherin-17, occludin and *ZO-1* purchased from TIB MOLBIOL GmbH (Berlin, Germany) and in the case of *Na^+^/K^+^-ATPase*, *PiT2*, *VDR*, *XPR1*, *VDR*, claudin-1 and claudin-15 synthesised by Thermo Fisher Scientific Inc. (Table 11). The reaction mixtures of 20 µL contained SensiFAST™ SYBR No-Rox Mix (BioCat, Heidelberg, Germany), 16 ng reverse-transcribed cDNA and 200 nmol/L specific primers. The PCR product amplification (3 min at 95 °C; 40 cycles of 10 s at 95 °C and 30 s at 60 °C) and analysis were conducted using a real-time PCR cycler (CFX96TM; Bio-Rad Laboratories GmbH). To determine the melt curve, the thermal profile began with an incubation of 10 min at 55 °C with a gradual increase in temperature (0.5 °C/10 s) to 95 °C. Calibration curves generated with cloned PCR fragment standards were used to determine the absolute copy numbers [88]. The specificity of the amplicons was verified by sequencing (Microsynth Seqlab GmbH, Göttingen, Germany) and using NCBI Blast (Bethesda, MD, USA) [89]. The expression abundance of genes of interest was normalised to *18S* as a constant expressed reference gene. Each reaction was carried out twice and included a no template control (NTC).

### 4.8. Intestinal Protein Expression of Na^+^/K^+^-ATPase, NaPiIIb and PiT1

The protein expression of Na^+^/K^+^-ATPase, NaPiIIb and PiT1 was examined exclusively in the ileal epithelium. This was done with regard to the results of the Ussing chamber experiments and the qPCR results, which indicate that the ileum is an important intestinal segment for P_i_ transport. For the abundance of NaPiIIb and PiT1, brush border membranes (BBM) were prepared using the method described by Wilkens et al. [88]. Crude membranes (CM) were isolated as described by Wilkens et al. [92] for the determining Na^+^/K^+^-ATPase protein expression. The protein concentrations of all cell fractions were measured with a commercial Bradford assay (Serva Electrophresis GmbH, Heidelberg, Germany). For PiT1, 10 µg of BBM were separated by 8.5% SDS-PAGE and transferred onto nitrocellulose membranes. The membranes were blocked in PBST and 5% fat-free milk powder for 1 h at RT. The PiT1 protein was detected in ileal BBM incubating with the diluted antibody (gift from V. Sorribas, Laboratory of Molecular Toxicology, University of Zaragoza, Zaragoza, Spain) in PBST over night at 4 °C. The immunoreactivity of the primary antibodies was detected using an HRP-coupled secondary antibody against rabbit (diluted 1:10,000). The bound antibodies were visualised using enhanced chemiluminescence (SuperSignal, Thermo Fisher Scientific Inc.) in accordance with the manufacturer’s protocol and a ChemiDoc system (Bio-Rad Laboratories GmbH). Immunoblot assays used for detecting of the expression of NaPiIIb and Na^+^/K^+^-ATPase proteins in caprine tissue were carried out as described elsewhere [3,93]. Total protein on immunoblots was stained with Indian ink. Densitometric measurements of proteins were performed using Image Lab 5.2.1 software (Bio-Rad Laboratories GmbH). In order to be able to semi-quantify the ileal NaPiIIb, PiT1 and Na^+^/K^+^-ATPase proteins, the band intensities for the examined proteins were normalised to the amount of total protein applied per lane.

### 4.9. Statistical Analysis

The sample size (minimum *n* 7/group) was calculated using the GPower software 3.1.9.4 based on metabolic data from previous work [3] with a statistical power of 0.8 and α error of 0.05. All data are given as means ± SEM. The statistical software GraphPad Prism version 8.0.1 for Windows (San Diego, CA, USA) [94] was used for data analysis. All data were tested for normal distribution using the Shapiro–Wilk test and analysed for significant differences using the unpaired Student’s *t*-test. The paired Student’s t-tests were performed for the differences of Gt and Isc values before and after adding of inhibitor substances (∆Isc, ∆Gt). Potential relationships between measured parameters were analysed by Pearson’s correlation and linear regression.

## Figures and Tables

**Table 1:** Mean daily intake of dry matter (DM), concentrate, phosphorus (P) and calcium (Ca) of young goats fed a low P diet. (Mean values with their standard errors; *n* = 7 animals).

Items	Control	P Reduction	*p*-Value
DM intake (g/day)	614 ± 10	528 ± 25	0.008
Concentrate intake (g/day)	555 ± 12	469 ± 29	0.017
P intake (g/day)	2.40 ± 0.05	0.53 ± 0.03	<0.001
Ca intake (g/day)	6.50 ± 0.13	5.42 ± 0.31	0.008

**Table 2:** Initial body weight and effects of a low phosphorus (P) diet on body weight gain and final body weight of young goats fed a low P diet. (Mean values with their standard errors; *n* = 7 animals).

Items	Control	P Reduction	*p*-Value
Initial body weight (kg) *	19.21 ± 0.75	18.93 ± 1.09	0.83
Final body weight (kg) †	25.29 ± 0.74	20.21 ± 0.93	0.001
Body weight gain (kg/d)	0.14 ± 0.01	0.03 ± 0.01	<0.001

* The initial body weight was determined at the beginning of the experimental feeding at ten weeks of age. † The final body weight was determined at the time of slaughter at 15–17 weeks of age.

**Table 3:** Inorganic phosphate (P_i_) and calcium (Ca) concentrations in saliva, ruminal and abomasal fluids of young goats fed a low phosphorus (P) diet. (Mean values with their standard errors; *n* = 7 animals).

Items	Control	P Reduction	*p*-Value
Plasma P_i_ (mmol/L)	2.09 ± 0.12	0.70 ± 0.04	<0.001
Plasma Ca (mmol/L)	3.16 ± 0.09	4.34 ± 0.17	<0.001
Saliva P_i_ (mmol/L)	31.77 ± 3.96	10.41 ± 0.83	<0.001
Saliva Ca (mmol/L)	0.42 ± 0.03	0.53 ± 0.02	0.01
Ruminal fluid P_i_ (mmol/L)	46.69 ± 6.11	6.07 ± 0.76	<0.001
Ruminal fluid Ca (mmol/L)	1.68 ± 0.11	5.75 ± 1.39	0.01
Abomasal fluid P_i_ (mmol/L)	39.02 ± 3.91	7.77 ± 0.78	<0.001
Abomasal fluid Ca (mmol/L)	17.83 ± 1.79	23.80 ± 3.48	0.15

**Table 4:** Inorganic phosphate (P_i_) and mannitol (Man) flux rates of duodenal and ileal epithelia of young goats fed a low phosphorus (P) diet. (Mean values with their standard errors; *n* = 7 animals).

Items	Control	P Reduction	*p*-Value
Duodenum			
P_i_ J_ms_	46.72 ± 5.69	48.85 ± 10.80	0.87
P_i_ J_sm_	32.92 ± 5.11	29.30 ± 3.09	0.56
P_i_ J_net_	13.80 ± 4.91	19.55 ± 10.59	0.63
Man J_ms_	26.31 ± 4.16	29.14 ± 6.87	0.73
Man J_sm_	26.13 ± 3.99	25.08 ± 3.38	0.84
Man J_net_	0.18 ± 3.52	4.06 ± 4.90	0.53
Ileum			
P_i_ J_ms_	215.49 ± 39.03	177.91 ± 45.56	0.54
P_i_ J_sm_	13.12 ± 2.49	15.44 ± 2.14	0.49
P_i_ J_net_	202.37 ± 40.09	162.47 ± 46.85	0.53
Man J_ms_	29.30 ± 5.51	33.77 ± 5.99	0.59
Man J_sm_	17.08 ± 1.95	20.70 ± 3. 01	0.33
Man J_net_	12.22 ± 4.48	13.07 ± 3.41	0.88

**Table 5:** Electrophysiological properties of duodenal and ileal epithelia of young goats fed a low phosphorus (P) diet. (Mean values with their standard errors; *n* = 7 animals).

Items	Control	P Reduction	*p*-Value
Duodenum			
I_sc_	0.61 ± 0.06	0.71 ± 0.04	0.20
G_t_	18.92 ± 1.83	18.05 ± 0.97	0.68
Ileum			
I_sc_	1.73 ± 0.29	1.12 ± 0.24	0.13
G_t_	17.18 ± 1.70	16.78 ± 1.25	0.85

I_sc_, short-circuit current in µeq/(cm^2^∙h); G_t_, tissue conductance in mS/cm^2^.

**Table 6:** Changes in tissue conductance (∆G_t_) and short-circuit current (∆I_sc_) after mucosal addition of Na^+^-arsenate and 2,4,6-triaminopyrimidine (TAP) in duodenum and ileum of young goats fed a low phosphorus (P) diet. (Mean values with their standard errors; *n* = 7 animals).

Items	Control	P Reduction	*p*-Value
Duodenum			
∆I_sc_ Na^+^-arsenate	0.31 ± 0.06	0.39 ± 0.03	0.25
∆I_sc_ TAP	−0.15 ± 0.03	−0.13 ± 0.03	0.59
∆G_t_ Na^+^-arsenate	4.91 ± 0.87	8.65 ± 2.13	0.13
∆Gt TAP	−3.96 ± 0.98	−3.03 ± 1.19	0.56
Ileum			
∆I_sc_ Na^+^-arsenate	−0.79 ± 0.21	−0.44 ± 0.18	0.23
∆I_sc_ TAP	−0.41 ± 0.17	−0.33 ± 0.10	0.69
∆G_t_ Na^+^-arsenate	8.42 ± 1.61	6.10 ± 1.93	0.37
∆Gt TAP	−1.69 ± 0.44	−2.17 ± 0.50	0.49

∆I_sc_, delta short-circuit current, difference between I_sc_ before and after inhibitor addition in µeq/(cm^2^∙h); ∆G_t_, delta tissue conductance, difference between G_t_ before and after inhibitor addition in mS/cm^2^.

**Table 7:** Relative mRNA expressions of proteins in the duodenum, mid jejunum and ileum of young goats fed a low phosphorus (P) diet, normalised to *18S*. (Mean values with their standard errors; *n* = 7 animals).

Items	Control	P Reduction	*p*-Value
Duodenum			
*Na^+^/K^+^-ATPase*	1.657 ± 0.318	1.615 ± 0.252	0.921
*NaPiIIb*	1.361 × 10^−6^ ± 5.762 × 10^−7^	6.360 × 10^−6^ ± 2.581 × 10^−6^	0.083
*PiT1*	0.010 ± 0.001	0.012 ± 0.003	0.606
*PiT2*	0.008 ± 0.004	0.010 ± 0.002	0.621
*VDR*	0.019 ± 0.002	0.018 ± 0.003	0.966
*XPR1*	0.089 ± 0.016	0.088 ± 0.012	0.959
*Cadherin* *-* *17*	1.494 ± 0.384	1.508 ± 0.162	0.974
*Claudin-1*	0.017 ± 0.003	0.015 ± 0.003	0.657
*Claudin-2*	0.228 ± 0.028	0.215 ± 0.048	0.824
*Claudin-12*	0.004 ± 0.001	0.005 ± 0.001	0.603
*Claudin-15*	0.104 ± 0.011	0.112 ± 0.019	0.712
*Occludin*	0.041 ± 0.006	0.042 ± 0.005	0.948
*ZO-1*	0.011 ± 0.002	0.010 ± 0.001	0.660
Mid jejunum			
*Na^+^/K^+^-ATPase*	2.925 ± 0.399	2.589 ± 0.208	0.468
*NaPiIIb*	0.158 ± 0.050	0.0831 ± 0.018	0.189
*PiT1*	0.011 ± 0.002	0.013 ± 0.002	0.537
*PiT2*	0.009 ± 0.001	0.008 ± 0.001	0.618
*VDR*	0.060 ± 0.009	0.050 ± 0.003	0.317
*XPR1*	0.020 ± 0.002	0.020 ± 0.002	0.948
*Cadherin-17*	2.197 ± 0.369	1.718 ± 0.119	0.240
*Claudin-1*	0.071 ± 0.008	0.073 ± 0.012	0.889
*Claudin-2*	0.759 ± 0.117	0.391 ± 0.088	0.027
*Claudin-12*	0.010 ± 0.002	0.009 ± 0.002	0.743
*Claudin-15*	0.788 ± 0.134	0.617 ± 0.076	0.287
*Occludin*	0.146 ± 0.030	0.111 ± 0.010	0.289
*ZO-1*	0.014 ± 0.002	0.011 ± 0.0005	0.215
Ileum			
*Na^+^/K^+^-ATPase*	0.911 ± 0.195	1.175 ± 0.123	0.274
*NaPiIIb*	0.006 ± 0.001	0.005 ± 0.002	0.858
*PiT1*	0.017 ± 0.003	0.025 ± 0.002	0.038
*PiT2*	0.014 ± 0.002	0.020 ± 0.002	0.046
*VDR*	0.007 ± 0.001	0.007 ± 0.001	0.890
*XPR1*	0.014 ± 0.002	0.017 ± 0.002	0.357
*Cadherin-17*	2.582 ± 0.425	3.478 ± 0.450	0.174
*Claudin-1*	0.013 ± 0.002	0.017 ± 0.003	0.277
*Claudin-2*	0.461 ± 0.088	0.510 ± 0.085	0.699
*Claudin-12*	0.009 ± 0.002	0.009 ± 0.001	0.990
*Claudin-15*	0.045 ± 0.012	0.040 ± 0.005	0.718
*Occludin*	0.136 ± 0.029	0.187 ± 0.032	0.254
*ZO-1*	0.020 ± 0.004	0.027 ± 0.003	0.164

*Na^+^/K^+^-ATPase*, Na^+^/K^+^-adenosine triphosphatase; *NaPiIIb*, Na^+^-dependent P_i_ transporter IIb; *PiT1*, Na^+^-dependent P_i_ transporter 1; *PiT2*, Na^+^-dependent P_i_ transporter 2; *XPR1*, xenotropic and polytropic retrovirus receptor 1; *ZO-1*, zonula occludens protein-1; *VDR*, vitamin D receptor.

**Table 8:** Relative amounts of Na^+^/K^+^-ATPase, NaPiIIb and PiT1 protein expression normalised to total protein amounts in the ileum of goats fed a phosphorus (P) reduced diet. (Mean values with their standard errors; *n* = 7 animals).

Items	Control	P Reduction	*p*-Value
Na^+^/K^+^-ATPase	0.119 ± 0.004	0.115 ± 0.005	0.61
NaPiIIb	0.040 ± 0.006	0.027 ± 0.005	0.11
PiT1	0.012 ± 0.002	0.007 ± 0.001	0.03

Na^+^/K^+^-ATPase, Na^+^/K^+^-adenosine triphosphatase; NaPiIIb, Na^+^-dependent P_i_ transporter IIb; PiT1, Na^+^-dependent P_i_ transporter 1.

**Table 9:** Components and composition of wheat straw and pelleted concentrate diets *.

Items	Wheat Straw	Control	P Reduction
Components (as fed) (g/kg)			
Soybean meal		68.0	68.0
Urea		24.5	24.5
Wheat starch		378	379
Beet pulp		399	399
Mineral-vitamin premix †		10.0	10.0
MgHPO₄ · 3 H₂O		9.3	-
MgO		-	2.2
NaH_2_PO_4_ · 2 H_2_O		9.7	0.4
NaCl		1.4	1.4
NaHCO_3_		-	5.0
CaCO_3_		14.3	14.3
Sipernat 22S ‡		41.8	52.3
Molasses		10.0	10.0
Soybean oil		34.0	34.0
Composition			
DM (g/kg)	904	880	884
Nutrients (g/kg DM)			
Crude ash	39.8	100	101
CP	25.4	165	169
ADFom	579	87.5	87.1
aNDFom	855	157	179
Crude fat	24.3	43.2	47.5
Urea	BDL	27.8	31.1
Ca	2.8	12.6	12.3
P	0.6	4.8	1.1
Vitamin D_3_ (IU/kg DM)	BDL	1000	1075
ME (MJ/kg DM)	8.3	12.8	12.9

BDL, below detection level; CP, crude protein; ME, metabolisable energy; ADFom, acid-detergent fibre expressed exclusive of residual ash; aNDFom, neutral-detergent fibre assayed with heat stable amylase expressed exclusive of residual ash. * Composition analysed by the Association of German Agricultural Investigation and Research Centre (VDLUFA). † Mineral-vitamin premix per kg: 0.2 g P; 12.1 g Ca; 1.7 g Na; 2.2 g Mg; 1,200,000 IU vitamin A; 120,000 IU vitamin D; 10,000 mg vitamin E; 675 mg vitamin K; 4960 mg iron; 6336 mg Zn; 501 mg Cu; 3000 mg Mn; 201 mg Co; 15 mg Se; 202 mg I. ‡ Sipernat 22S (Evonik Industries AG, Essen, Germany) is a fine particle salica used in the food and feed industry as a highly absorbent carrier substance, flow regulator, anti-caking and anti-dusting agent.

**Table 10:** Primers and probes used for TaqMan™ assays.

Genes	Primers and Probes (5′ → 3′)	Accession Number	References
*18S*	Forward: AAAAATAACAATACAGGACTCTTTCG Reverse: GCTATTGGAGCTGGAATTACCG 6FAM-TggAATgAgTCCACTTTAAATCCTTCCgC--BBQ	KY129860	[90]
*Claudin-2*	Forward: CCAAAGACAGAGTGGCGGT Antisense: TCAAATTTCATGCTGTCAGGCAC FAM-TCCTGGGCTTCATCCCYGTTGC-BBQ	XM_005700206.3	[72]
*Claudin-12*	Sense: GCTGCTCTGCCTCATCGG Antisense: GCAGCCYGCACTATTGACCA FAM-TGTGTAACACGGCCTTCAGGTCCTC-BBQ	XM_005678898.3	[72]

**Table 11:** Primers used for SYBR Green assays.

Genes	Primers (5′ → 3′)	Accession Number	References
*Na^+^/K^+^-ATPase*	Sense: TGGAACTCGGAGAAGAAGGA Antisense: GCCACTCGGTCCTGATATGT	XM_005690616.3	[3]
*NaPiIIb*	Sense: CGGTCCAAAACAAAAGTATGATCAAG Reverse: AGCCAAAGGGGTAAGGGAA	XM_005681484.3 to XM_013964569.2	[3]
*PiT1*	Sense: ATTCATCCTCCGTAAGGCAGATC Antisense: CAGCAATGGTGCTCCAGTATACA	XM_018055327.1	[3]
*PiT2*	Forward: CCAATCTCGGGGACTCACTG Reverse: GGAACGGGGTCCTCCTTTTT	XM_018041866.1 to XM_018041872.1	This study
*VDR*	Forward: GCACTTCCTTACCTGACCCC Reverse: CCGCTTGAGGATCATCTCCC	XM_004007435.1	[91]
*XPR1*	Forward: AATGCCGATGATCAGACGCT Reverse: AGCCTTGGATTGAGAAGCGA	XM_018060672.1	This study
*Cadherin-17*	Sense: CACCCTTTTGGTCATCGGTAT Antisense: CATCAGTTTCTCAGAGGCTTGACT	XM_018058228.1	[72]
*Claudin-1*	Forward: TTCATCCTGGCGTTTCTGGG Reverse: GTTGCTTGCAGAGTGCTGTT	XM_005675123.3	This study
*Claudin-15*	Forward: AGGGACTTCTTCGACCCCTT Reverse: CGTTATCACGGGGGCTTTGT	XM_013974836.2 to XM_018040880.1	This study
*Occludin*	Sense: CTCGTCTGGATAAAGAACTGGATGA Antisense: CTCGTCTGGATAAAGAACTGGATGA	XM_018065677.1 to XM_018065681.1	[72]
*ZO-1*	Sense: CTCAGTACAGCCAGGGTGCT Reverse: TCCGGTTTGGACACTAATGAGTT	XM_018066114.1 to XM_018066118.1	[72]

## Data Availability

The data presented in this study are available on request from the corresponding author.

## Abbreviations

1,25-(OH)_2_D_3_	1,25-dihydroxy-vitamin D_3_
25-(OH)D_3_	25-hydroxy-vitamin D_3_
AJ	Adherens-junctions
BBM	Brush border membranes
Ca	Calcium
CP	Crude protein
DM	Dry matter
K^+^	Potassium
ME	Metabolisable energy
N	Nitrogen
Na^+^	Sodium
Na^+^/K^+^-ATPase	Sodium-potassium adenosine triphosphatase
NaPiIIb	Type IIb sodium-dependent phosphate transporter
P	Phosphorus
P_i_	Inorganic phosphate
RT	Room temperatur
PiT1/2	Type III sodium-dependent phosphate transporter 1/2
TJ	Tight-junctions
VDR	Vitamin D receptor
XPR1	xenotropic and polytropic retrovirus receptor 1
ZO-1	Zonula occludens protein-1
